# Bronchoscopy-guided non-capping decannulation pathway versus conventional capping trial in patients with prolonged tracheostomy: a retrospective comparative cohort study

**DOI:** 10.3389/fmed.2026.1825058

**Published:** 2026-05-15

**Authors:** Rundi Gao, Guofeng Wang, Ping Ni, Jiayan Zhong, Liyan Zhang, Minfang Wang, Yajun Mao, Huihua Hong

**Affiliations:** 1 Department of Pulmonary and Critical Care Medicine, The First Affiliated Hospital of Zhejiang Chinese Medical University (Zhejiang Provincial Hospital of Chinese Medicine), Hangzhou, China; 2 Nursing Department, The First Affiliated Hospital of Zhejiang Chinese Medical University (Zhejiang Provincial Hospital of Chinese Medicine), Hangzhou, China; 3 Department of Public Health & Hospital Infection Control, The First Affiliated Hospital of Zhejiang Chinese Medical University (Zhejiang Provincial Hospital of Chinese Medicine), Hangzhou, China; 4 Comprehensive Rehabilitation Department, The First Affiliated Hospital of Zhejiang Chinese Medical University (Zhejiang Provincial Hospital of Chinese Medicine), Hangzhou, China

**Keywords:** bronchoscopy, capping trial, decannulation, prolonged tracheostomy, retrospective cohort

## Abstract

**Background:**

Tracheostomy decannulation is commonly guided by capping trials that assess functional tolerance but do not directly evaluate airway anatomy. Whether a bronchoscopy-guided, non-capping decannulation pathway offers comparable safety with efficiency remains uncertain.

**Methods:**

We conducted a retrospective comparative cohort study of adults with prolonged tracheostomy (≥ 4 weeks) between 2023 and 2025 at a tertiary center. Patients underwent either a bronchoscopy-guided non-capping pathway (*n* = 23) or a conventional capping trial (*n* = 58). The primary outcome was decannulation failure, defined as having a tracheostomy tube in place at hospital discharge. Secondary outcomes included time to decannulation, reinsertion within 72 h, infectious complications, and length of stay. Multivariable and sensitivity analyses were performed to address baseline imbalances and rare events.

**Results:**

Eighty-one patients were analyzed. The non-capping group was older and had lower hemoglobin levels. Decannulation failure occurred in 4.3% of patients managed with the non-capping pathway and 20.7% with conventional capping (*p* = 0.141). Reinsertion within 72 h occurred in 1 and 4 patients, respectively. All reinsertion cases ultimately had a tracheostomy tube in place at discharge. A shorter time to successful decannulation (adjusted HR 1.94, 95% CI 1.09–3.48; *p* = 0.03) and a lower number of infectious episodes (*p* = 0.002) were observed in the non-capping pathway.

**Conclusion:**

In this single-center observational study, the non-capping pathway was associated with a shorter time to decannulation without an apparent increase in short-term safety events. These findings suggest a direct anatomical non-capping pathway may be a feasible alternative, although this requires further validation.

## Introduction

Tracheostomy is commonly performed in patients requiring prolonged mechanical ventilation or airway protection. However, prolonged cannulation is associated with substantial morbidity, including airway injury, infection, and delayed rehabilitation (1–3). Timely and safe decannulation is therefore a critical milestone, but it remains challenging, particularly in patients with prolonged tracheostomy and difficult weaning trajectories.

In current clinical practice, decannulation decisions primarily rely on functional tolerance testing, most commonly through tracheostomy capping (4). This approach infers airway patency indirectly based on physiological responses rather than direct anatomical assessment (5, 6). However, this “blind” strategy evaluates global respiratory reserve but may miss occult structural abnormalities—such as granulation tissue, tracheomalacia, or vocal fold dyskinesia—until clinical deterioration or decannulation failure occurs. Furthermore, the capping process itself may impose additional resistive loads, potentially precipitating acute distress or secretion retention in patients with limited reserve, unnecessarily delaying decannulation and prolonging hospitalization (7).

In contrast, flexible bronchoscopy allows real-time evaluation of airway anatomy and dynamic collapse. Although international consensus increasingly recommends endoscopic assessment as an important component of the decannulation process (1, 8, 9), its application remains predominantly reactive, typically reserved for failure cases of conventional capping trials. A recent pilot investigation provided preliminary evidence for the feasibility of a single-stage bronchoscopic decannulation protocol (10). However, no comparative clinical studies have evaluated whether this anatomy-driven approach improves clinical outcomes compared with conventional capping strategies.

In this retrospective cohort study, we evaluated two decannulation pathways in clinical practice: a bronchoscopy-guided, non-capping decannulation pathway and a conventional capping trial. Our study aimed to explore the feasibility and short-term safety of this approach. We hypothesized that this anatomy-driven pathway may accelerate the decannulation trajectory without increasing failure risk and may be associated with fewer complications. By shifting the clinical focus from subjective physiological tolerance to direct visual assessment, this research aims to explore a more individualized and potentially streamlined clinical pathway for patients with prolonged tracheostomy.

## Methods

### Study design and setting

This retrospective comparative cohort study was conducted at the Respiratory Rehabilitation and Comprehensive Rehabilitation Departments of The First Affiliated Hospital of Zhejiang Chinese Medical University (Hangzhou, China). The study was approved by the Institutional Ethics Committee (2025-KLS-897-01), which waived informed consent due to the retrospective design. The study was conducted in accordance with the Declaration of Helsinki and relevant local regulations.

### Study population and follow-up

We included adult patients (≥18 years) with prolonged tracheostomy (≥4 weeks) who underwent decannulation assessment between 1 January 2023 and 30 September 2025. As a retrospective study, sample size was not pre-specified by power calculation. Eligible patients were clinically stable, defined as hemodynamic stability without vasopressors, low-flow oxygen requirement (FiO₂ ≤ 40%), controlled infection, adequate airway protection and secretion clearance, assessed by effective cough (defined as the ability to clear secretions through the tracheostomy stoma) and a suction frequency of <8 times per 24 h.

These criteria were applied consistently across both pathways to standardize baseline readiness for decannulation assessment. Patients with irreversible contraindications to decannulation, contraindications to bronchoscopy, or insufficient documentation to determine eligibility for the decannulation pathway were excluded. In addition, a small number of otherwise eligible patients did not undergo decannulation assessment due to patient or family preference (e.g., concerns regarding reinsertion risk).

Patients were categorized into the conventional capping or bronchoscopy-guided non-capping group based on the predominant clinical pathway of the admitting department, representing a comparison of two clinical pathways. Although implemented in different departments, both pathways operated within a shared institutional framework, including a multidisciplinary team (MDT), standardized decannulation readiness criteria, and common protocols for airway management and monitoring. Tracheostomy tube diameter was determined by clinical judgment and was not used to guide pathway allocation. Patients were followed until hospital discharge or death, with a mandatory 72-h observation period after decannulation.

### Decannulation pathways

The conventional capping group underwent a standardized pathway consisting of replacement of the existing plastic tracheostomy tube with a metal tracheostomy tube to facilitate capping, followed by a 24–48 h capping period. This practice reflects routine clinical workflow in our institution, where metal tubes are preferred during capping due to their lower airflow resistance and ease of occlusion. Pathway completion was defined as successful tolerance of full tube occlusion throughout the planned capping period without respiratory distress, oxygen desaturation. If these criteria were met, decannulation was performed. Decannulation intolerance in this group was defined as inability to complete the planned capping period due to the above clinical instability, resulting in protocol discontinuation and reassessment.

The non-capping group underwent an anatomy-driven, non-capping pathway. Flexible bronchoscopy (BF-290; Olympus, Tokyo, Japan) was performed via both stomal and transnasal routes under topical anesthesia with minimal sedation, maintaining spontaneous breathing. Pathway completion was defined as fulfillment of predefined criteria permitting immediate decannulation, including: (1) anatomic patency: ≥50% cross-sectional area, based on previously published criteria (8); (2) dynamic stability: absence of severe tracheomalacia (expiratory collapse < 50%) or obstructing granulation (<30% lumen). When criteria were satisfied, the plastic tracheostomy tube was removed under direct bronchoscopic visualization during spontaneous breathing. Airway patency and dynamic collapse were monitored for at least 3 min. Clinical markers of intolerance included inspiratory stridor, tachypnea (respiratory rate > 30 breaths/min), oxygen desaturation (SpO₂ < 90%), or a ≥20% increase in heart rate from baseline. If no adverse signs occurred, the stoma was dressed. Decannulation intolerance was defined as failure to meet the predefined anatomical or clinical criteria, including identification of airway abnormalities or physiological instability that precluded immediate tube removal and required postponement or intervention.

### Outcomes

The primary outcome was decannulation failure, defined as having a tracheostomy tube in place at hospital discharge, reflecting a pathway-dependent process outcome.

Secondary outcomes included: (1) time to successful decannulation: the interval (in days) from the initiation of the decannulation assessment (Time Zero) to the definitive removal of the tracheostomy tube without requiring reinsertion within 72 h; (2) reinsertion of a tracheostomy tube within 72 h after decannulation as a short-term safety outcome; (3) infectious complications: the new-onset respiratory or systemic infections requiring initiation or escalation of antimicrobial therapy, confirmed by radiologic, laboratory, or microbiological findings as documented in the medical record. These events were recorded during the decannulation assessment period; (4) healthcare utilization: length of hospital stay (LOS) during the index admission. Additional safety markers included decannulation intolerance (inability to complete the assigned protocol), in-hospital mortality, and total tracheostomy duration.

### Statistical analysis

Normality of continuous variables was assessed by histograms and the Shapiro–Wilk test. Normally distributed data are presented as mean ± standard deviation and were compared using Student’s t-test. Non-normally distributed variables are presented as median with interquartile range (IQR) and were compared using the Mann–Whitney *U* test. Categorical variables are presented as counts and percentages and were compared using Fisher’s exact test, given the relatively small sample size.

The association between decannulation pathway and decannulation failure was evaluated using logistic regression. Multivariable models adjusted for age, hemoglobin, and GCS-M score. To prevent overfitting, this variable selection was maintained for standard adjusted models. Due to rare events, Firth’s penalized regression was used for sensitivity analysis. Results are reported as odds ratios (ORs) with 95% confidence intervals (CIs). To account for confounding due to non-randomized allocation, inverse probability of treatment weighting (IPTW)-weighted Firth penalized logistic regression based on propensity scores was applied for the primary outcome, given the limited number of events and potential quasi-complete separation. The propensity score was estimated using five covariates (age, hemoglobin, GCS-M, COPD, and pulmonary infection). Stabilized weights were calculated and truncated at the 1st and 99th percentiles to reduce the influence of extreme weights. Covariate balance was assessed via absolute standardized mean differences (SMDs), with <0.1 indicating adequate balance.

Time to successful decannulation was analyzed using Kaplan–Meier curves and Cox proportional hazards models with adjustment for the same prespecified covariates. The proportional hazards assumption was verified. Given the limited number of outcome events, time-to-event and secondary analyses were interpreted as supportive rather than confirmatory.

Infectious complications were analyzed using analogous unadjusted, multivariable, Firth-adjusted, and IPTW-weighted models, using the same covariate structure as for the primary outcome.

All analyses were conducted in R (version 4.4.2). Two-sided *p* < 0.05 was considered statistically significant.

## Results

### Study population

**Figure 1:**
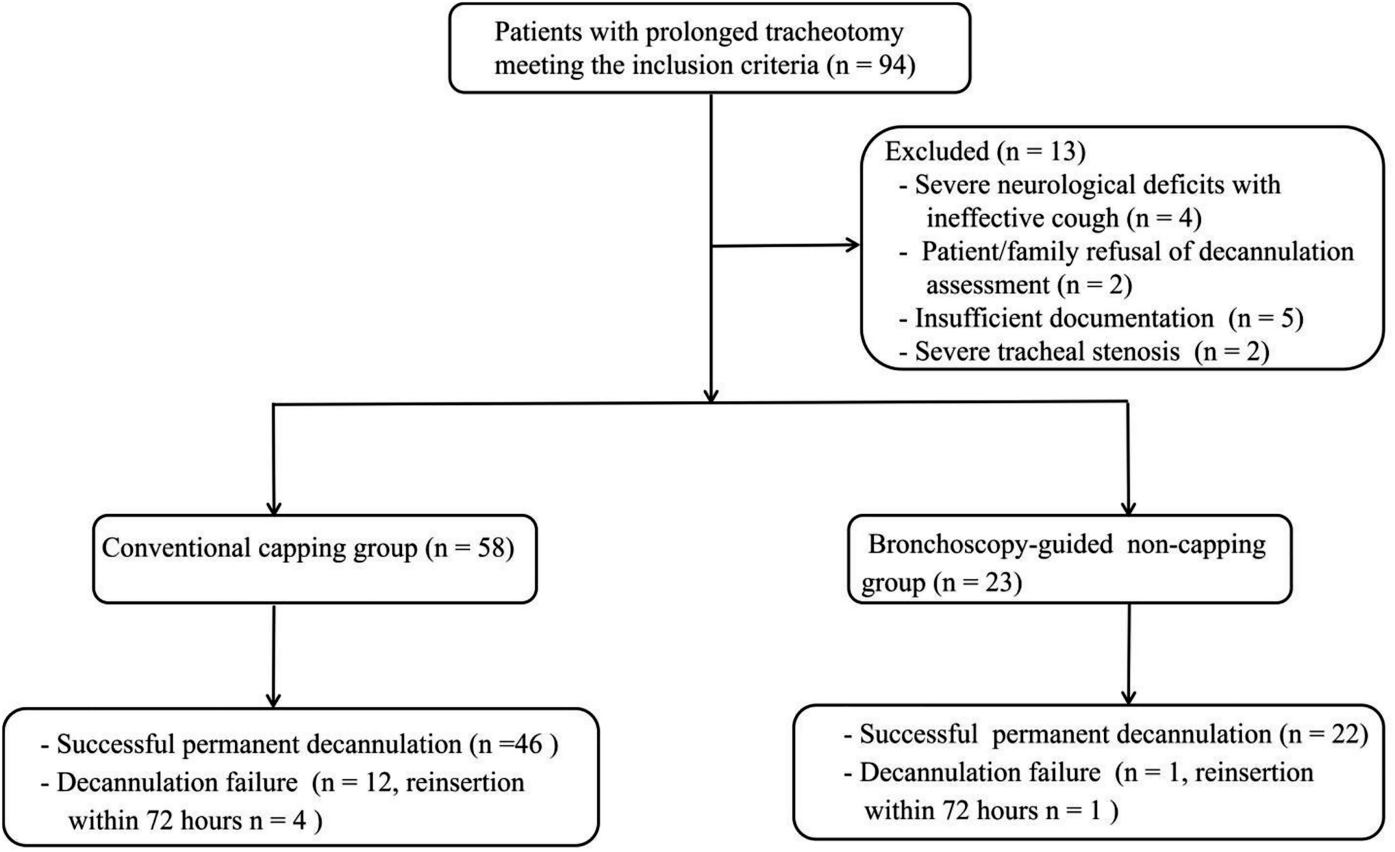
Flow diagram of patient screening, exclusion, and cohort allocation according to decannulation pathway.

A total of 94 patients with prolonged tracheostomy were screened for decannulation eligibility, of whom 13 (13.8%) were excluded for predefined reasons (Figure 1). The final analytic cohort consisted of 81 patients: 58 patients (71.6%) managed with conventional capping trial, and 23 patients (28.4%) underwent the bronchoscopy-guided non-capping pathway.

Successful permanent decannulation was achieved in 46/58 (79.3%) patients in the conventional group and 22/23 (95.7%) in the non-capping group. Early reinsertion within 72 h occurred in four patients and one patient, respectively. No patients were excluded due to missing baseline clinical data among those who met eligibility criteria.

### Baseline characteristics

Baseline characteristics are summarized in Table 1. Patients in the non-capping group were older (median age 77.0 vs. 64.0 years, *p* < 0.001) and had lower hemoglobin levels (median 88.0 vs. 104.0 g/L, *p* = 0.003). The non-capping group also demonstrated a higher GCS motor score (median GCS-M 6.0 vs. 6.0, *p* = 0.046). Other clinical and laboratory parameters did not differ significantly between groups.

**Table 1:** Baseline characteristics of patients according to decannulation pathway.

Variable	Conventional capping (*n* = 58)	Non-capping (*n* = 23)	*p*
Demographics			
Age (years) (median [IQR])	64.00 [54.25, 70.00]	77.00 [71.00, 80.00]	**<0.001**
Gender (%)			
Male	35 (60.3)	15 (65.2)	0.878
Female	23 (39.7)	8 (34.8)	
Neurological status			
GCS score (Verbal) (median [IQR])	4.00 [4.00, 4.00]	4.00 [4.00, 4.00]	0.199
GCS score (Motor) (median [IQR])	6.00 [5.00, 6.00]	6.00 [6.00, 6.00]	0.046
Comorbidities			
COPD (%)			
No	51 (87.9)	18 (78.3)	0.449
Yes	7 (12.1)	5 (21.7)	
Pulmonary infection (%)			
No	4 (6.9)	2 (8.7)	1.000
Yes	54 (93.1)	21 (91.3)	
Respiratory failure (%)			
No	28 (48.3)	9 (39.1)	0.619
Yes	30 (51.7)	14 (60.9)	
AF (%)			
No	52 (89.7)	21 (91.3)	1.000
Yes	6 (10.3)	2 (8.7)	
HF (%)			
No	52 (89.7)	16 (69.6)	0.059
Yes	6 (10.3)	7 (30.4)	
CAD (%)			
No	49 (84.5)	15 (65.2)	0.106
Yes	9 (15.5)	8 (34.8)	
Stroke (%)			
No	30 (51.7)	15 (65.2)	0.393
Yes	28 (48.3)	8 (34.8)	
Gastrointestinal bleeding (%)			
No	54 (93.1)	17 (73.9)	**0.046**
Yes	4 (6.9)	6 (26.1)	
Renal insufficiency (%)			
No	48 (82.8)	18 (78.3)	0.879
Yes	10 (17.2)	5 (21.7)	
Malignancy (%)			
No	53 (91.4)	16 (69.6)	**0.032**
Yes	5 (8.6)	7 (30.4)	
2DM (%)			
No	47 (81.0)	20 (87.0)	0.757
Yes	11 (19.0)	3 (13.0)	
Surgery (%)			
No	43 (74.1)	11 (47.8)	**0.045**
Yes	15 (25.9)	12 (52.2)	
Trauma (%)			
No	39 (67.2)	17 (77.3)	0.548
Yes	19 (32.8)	5 (22.7)	
VTE (%)			
No	21 (36.2)	10 (43.5)	0.724
Yes	37 (63.8)	13 (56.5)	
Laboratory findings			
White blood cell (10^9/L) (median [IQR])	6.85 [5.40, 8.95]	6.30 [4.70, 7.60]	0.088
Hemoglobin (g/L) (median [IQR])	104.00 [96.00, 112.75]	88.00 [83.00, 103.50]	**0.003**
PLT (10^9/L) (median [IQR])	232.50 [194.25, 297.50]	222.00 [157.50, 272.00]	0.160
Albumin (g/L) (median [IQR])	34.05 [32.00, 36.27]	34.20 [31.55, 36.80]	0.867
Cr (μmol/L) (median [IQR])	45.50 [38.25, 59.50]	48.00 [42.00, 68.00]	0.148
D-dimer (mg/L FEU) (median [IQR])	0.92 [0.54, 1.49]	0.80 [0.60, 1.65]	0.946
pH (median [IQR])	7.42 [7.39, 7.45]	7.42 [7.40, 7.44]	0.979
PaO2 (mmHg) (median [IQR])	117.00 [96.13, 148.75]	135.00 [89.25, 173.00]	0.579
PaCO2 (mmHg) (median [IQR])	43.65 [37.18, 46.27]	43.20 [39.25, 47.90]	0.216
Heart rate (bpm) (median [IQR])	83.50 [75.00, 89.00]	84.00 [78.00, 88.00]	0.496
CRP (mg/L) (median [IQR])	8.09 [4.08, 14.64]	7.47 [3.06, 12.98]	0.722

Data are presented as median [interquartile range] for continuous variables and as number (percentage) for categorical variables. Continuous variables were compared using the Mann–Whitney *U* test. Categorical variables were compared using the Chi-square test or Fisher’s exact test, as appropriate. Bold *p*-values indicate statistical significance at the *p* < 0.05 level. GCS, Glasgow Coma Scale (V, Verbal; M, Motor); CRP, C-reactive protein; PLT, platelet count; Cr, creatinine; PaO2, arterial partial pressure of oxygen; PaCO2, arterial partial pressure of carbon dioxide; COPD, chronic obstructive pulmonary disease; RF, respiratory failure; AF, atrial fibrillation; HF, heart failure; CAD, coronary artery disease; 2DM, type 2 diabetes mellitus; VTE, venous thromboembolism.

### Decannulation-related outcomes

Decannulation-related outcomes are presented in Table 2. Decannulation intolerance occurred in 23/58 patients (39.7%) in the conventional capping group and 9/23 patients (39.1%) in the non-capping group (*p* = 1.000). Decannulation failure was observed in 12 patients (20.7%) in conventional capping compared with 1 patient (4.3%) in the non-capping group (*p* = 0.141), representing an absolute risk reduction of 16.4%. Reinsertion within 72 h occurred in 4 patients in the conventional group and 1 patient in the non-capping group, all of these patients ultimately had a tracheostomy tube in place at hospital discharge.

**Table 2:** Clinical outcomes and time-related variables according to decannulation pathway.

Variable	Conventional capping	Non-capping	*p*
Clinical outcomes, *n* (%)	58	23	
Reinsertion within 72 h	4 (6.9)	1 (4.3)	1.000
Decannulation failure	12 (20.7)	1 (4.3)	0.141
In-hospital mortality	0 (0.0)	0 (0.0)	NA
Decannulation Intolerance	23 (39.7)	9 (39.1)	1.000
Time and complications, median [IQR]			
Time to decannulation (days)	7.50 [4.00, 17.25]	0.00 [0.00, 17.00]	0.029
Total tracheostomy duration (days)	97.50 [71.75, 131.00]	105.00 [44.50, 215.50]	0.549
Length of hospital stay (days)	119.00 [53.00, 192.00]	96.50 [44.50, 121.75]	0.097
No. of infectious episodes during decannulation	1.00 [0.00, 1.00]	0.00 [0.00, 0.00]	0.002

Data are presented as median [IQR] or *n* (%). *p*-values were calculated using the Mann–Whitney *U* test for continuous variables and Fisher’s exact test for categorical variables. IQR, interquartile range; NA, not applicable. Bold *p*-values indicate statistical significance (*p* < 0.05).

Among patients undergoing the decannulation process, the median time from assessment initiation to tube removal was shorter in the non-capping group (median 0.0 vs. 7.5 days; *p* = 0.029). Total tracheostomy duration and LOS were comparable between groups.

A lower number of infectious episodes were observed in patients managed with the non-capping pathway during the decannulation assessment period (median 0 vs. 1; *p* = 0.002). No in-hospital mortality occurred.

### Multivariable and sensitivity analyses of decannulation failure

The results of the multivariable and sensitivity analyses are presented in Table 3. In multivariable logistic regression adjusting for prespecified covariates (age, hemoglobin level, and GCS motor score), the non-capping pathway was not independently associated with decannulation failure (OR 0.16; 95% CI 0.01–1.06; *p* = 0.11). None of the included covariates demonstrated a statistically significant association with decannulation failure.

**Table 3:** Multivariable and sensitivity analyses of decannulation failure.

Analytic model	OR/HR	95% CI (lower)	95% CI (upper)	*p*-value
Crude logistic regression	0.17	0.01	0.97	0.10
Adjusted logistic regression	0.16	0.01	1.06	0.11
Firth penalized logistic regression	0.25	0.02	1.29	0.10
IPTW-weighted Firth logistic regression	0.09	0.001	0.81	0.03
Cox proportional hazards (unadjusted)	2.01	1.20	3.36	0.01
Cox proportional hazards (adjusted)	1.94	1.09	3.48	0.03

For Logistic/Firth models, OR < 1 indicates a lower risk of failure. For Cox proportional hazards models, HR > 1 indicates a shorter time to successful decannulation. OR, odds ratio; HR, hazard ratio; CI, confidence interval; IPTW, inverse probability of treatment weighting; GCS-M, Glasgow Coma Scale motor score. *To prevent overfitting given the limited number of failure events, standard adjusted models (logistic, Firth, and Cox) included three prespecified covariates: age, hemoglobin level, and GCS-M score. For the IPTW-weighted Firth model, the propensity score was estimated using a broader set of covariates (age, hemoglobin, GCS-M, COPD, and pulmonary infection) to maximize baseline balance, while the Firth penalty addressed the rare failure event in the non-capping group. All adjusted and weighted analyses were exploratory in nature.

Firth’s penalized logistic regression was applied as a sensitivity analysis to reduce small-sample bias. Firth’s penalized regression yielded similar directionally consistent results (OR 0.25; 95% CI 0.02–1.29; *p* = 0.10).

**Figure 2:**
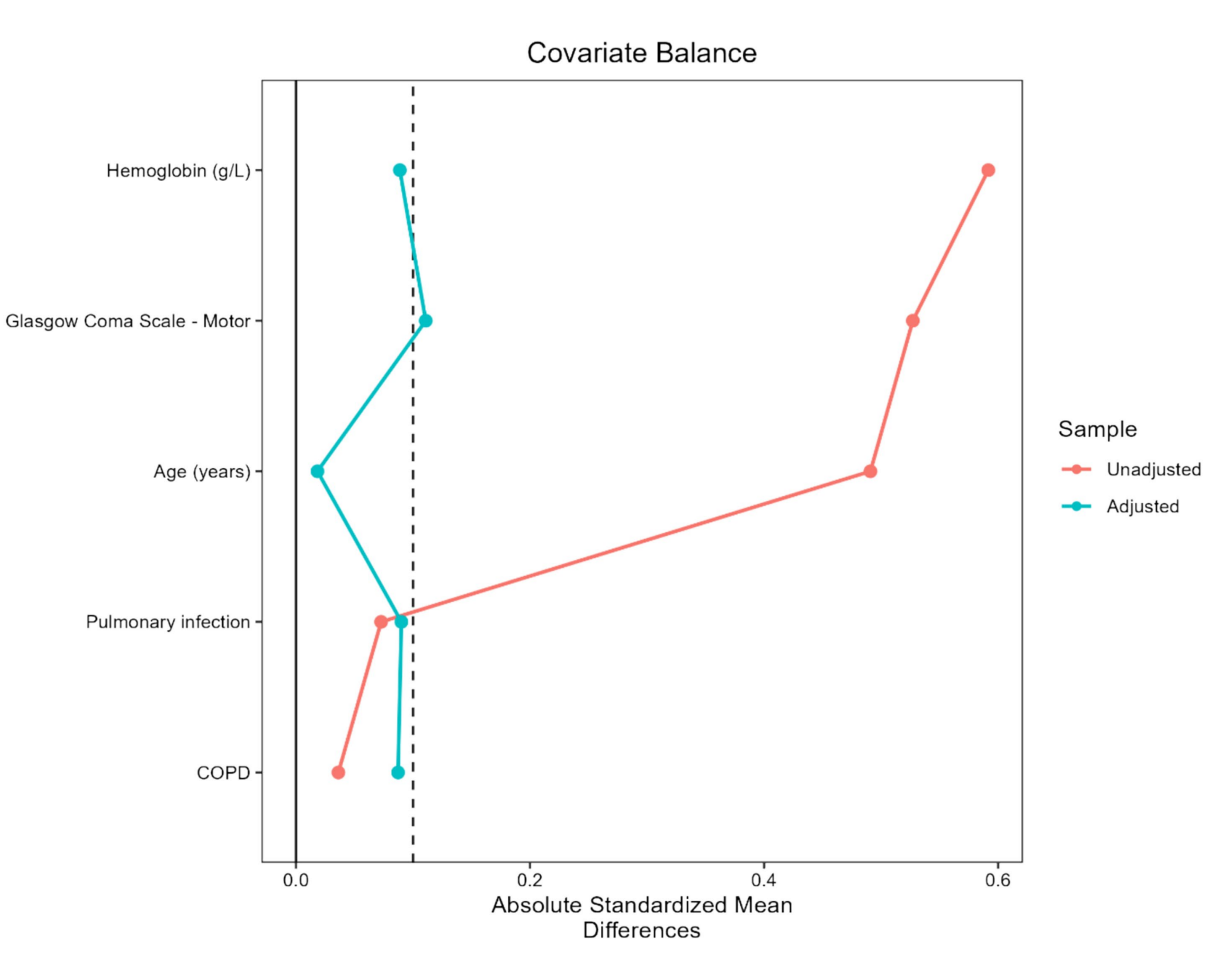
Covariate balance before and after inverse probability of treatment weighting (IPTW). Love plot illustrating the absolute standardized mean differences (SMDs) of baseline covariates between the conventional capping and non-capping groups before (unadjusted, red/orange) and after (adjusted, blue/teal) the application of truncated stabilized weights. The plot includes all five covariates incorporated into the propensity score model: age, hemoglobin, Glasgow Coma Scale-Motor (GCS-M) score, COPD, and pulmonary infection. Following IPTW adjustment, the SMDs for all included covariates were substantially reduced to approximately 0.1 or below, indicating adequate covariate balance between the two study groups.

**Figure 3:**
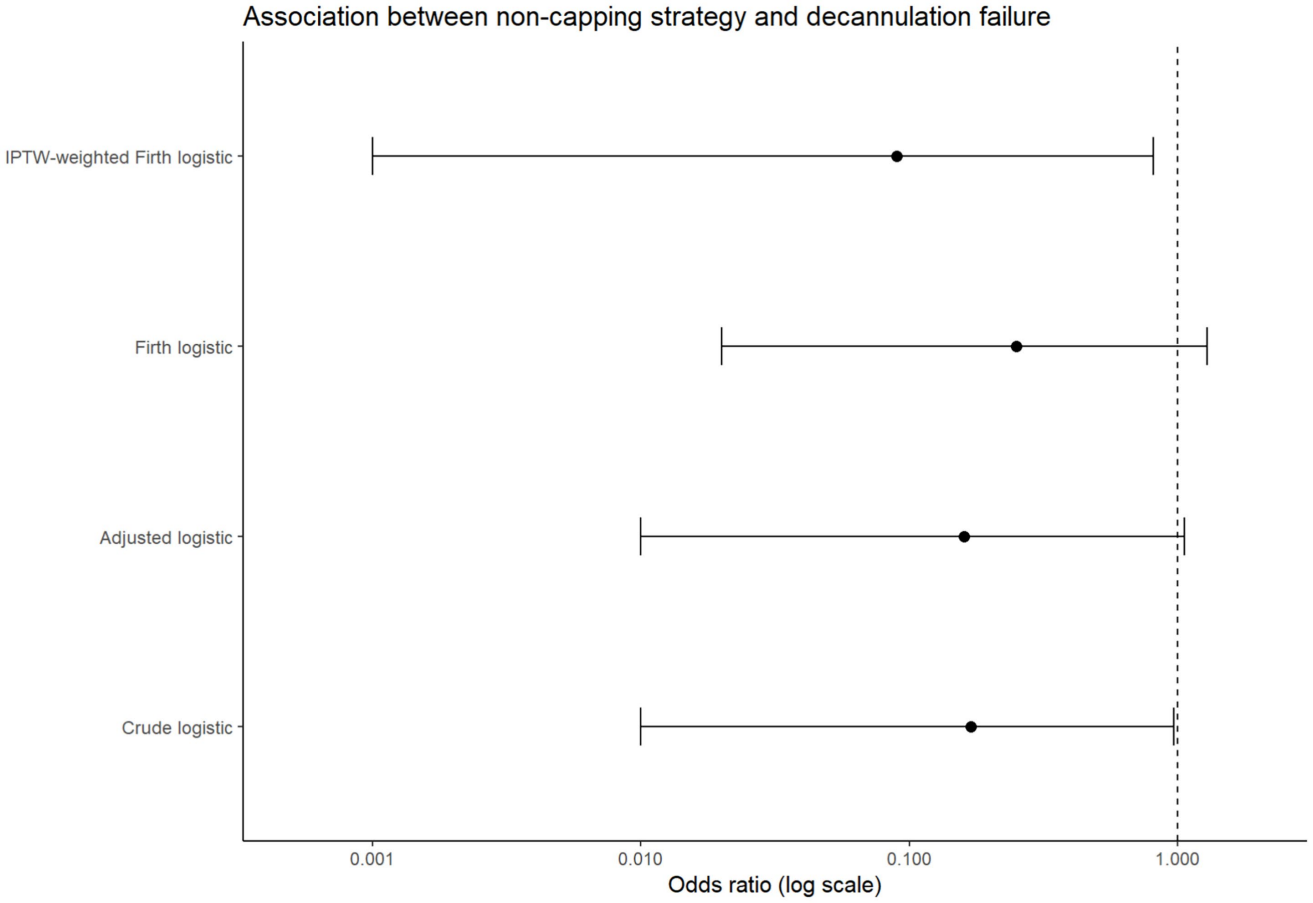
Association between the non-capping pathway and decannulation failure across multiple analytic models. Forest plot showing odds ratios (ORs) and 95% confidence intervals (CIs) for decannulation failure comparing the non-capping pathway with conventional capping. Estimates are derived from four models: unadjusted (crude) logistic regression, multivariable-adjusted logistic regression (adjusted for age, hemoglobin level, and Glasgow Coma Scale–Motor score), Firth’s penalized logistic regression, and IPTW-weighted Firth logistic regression (utilizing truncated stabilized weights derived from a propensity score model incorporating five core covariates). The vertical dashed line indicates an odds ratio of 1.0.

To further address potential confounding by indication, baseline covariates were balanced after weighting (all standardized mean differences < 0.1) (Figure 2; Supplementary Figure 2; Supplementary Table 2). In the IPTW-weighted Firth analysis, the absolute number of failure events was lower in the non-capping pathway, with model-based estimates showing a similar direction of association (OR 0.09; 95% CI 0.001–0.81; *p* = 0.03) (Figure 3), although these estimates were imprecise due to the limited number of events and wide confidence intervals. Associations with infectious complications showed broadly consistent patterns across multivariable, penalized, and propensity-weighted models (Supplementary Table 1 and Supplementary Figure 1; covariate balance is presented in Figure 2).

### Time-to-event analysis

**Figure 4:**
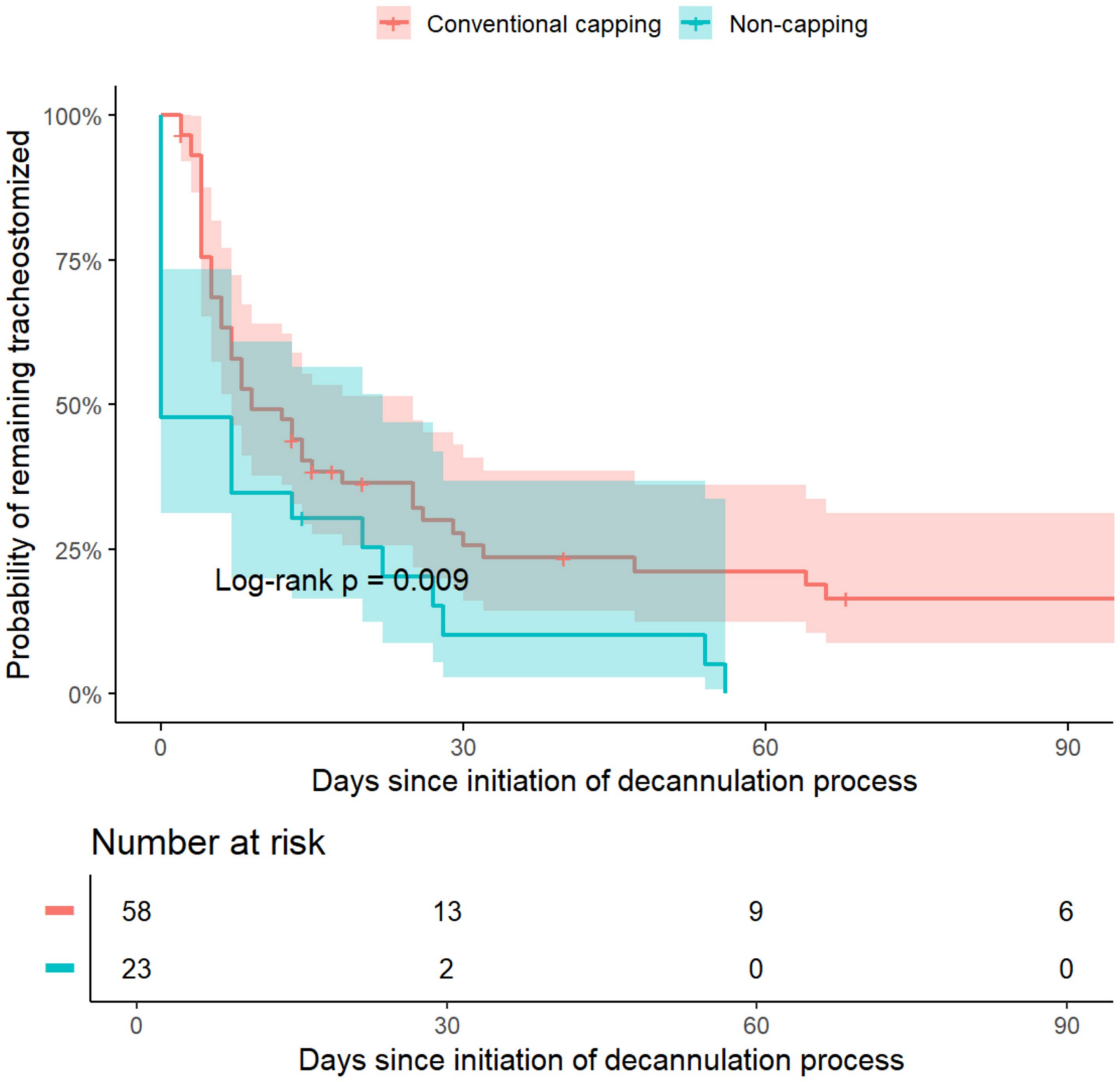
Kaplan–Meier curves for time to successful decannulation. Kaplan–Meier curves depicting time from initiation of the decannulation process to successful decannulation stratified by decannulation pathway. Patients experiencing decannulation failure were censored at hospital discharge. Shaded areas represent 95% confidence intervals. The non-capping pathway showed a shorter time to successful decannulation compared with conventional capping (log-rank *p* = 0.009).

An exploratory time-to-event analysis was conducted to evaluate the association between decannulation pathway and time to successful decannulation. The median time to successful decannulation was shorter in the non-capping pathway (0.0 vs. 7.5 days). Kaplan–Meier analysis showed showed a similar direction (log-rank *p* = 0.009; Figure 4). Separation of survival curves was observed early after initiation of the decannulation pathway. In Cox proportional hazards models adjusted for prespecified covariates, the non-capping pathway showed a higher estimated hazard of successful decannulation compared with conventional capping (adjusted HR 1.94; 95% CI 1.09–3.48; *p* = 0.03; Table 3). Assessment of the proportional hazards assumption using Schoenfeld residuals did not reveal a significant global violation (global test *p* = 0.15).

## Discussion

In this retrospective cohort study, we compared two clinical decannulation pathways in routine clinical practice for patients with prolonged tracheostomy. Decannulation failure, defined as having a tracheostomy tube in place at hospital discharge, occurred less frequently in the bronchoscopy-guided non-capping group, but this difference did not reach statistical significance. The non-capping pathway was associated with a shorter time to decannulation without an apparent increase in short-term safety events, as reflected by similar rates of reinsertion within 72 h. A lower number of infectious episodes was observed. Together, these findings suggest that the bronchoscopy-guided pathway may be a feasible alternative within a multidisciplinary care setting, although these observations should be interpreted cautiously.

The definition of decannulation outcomes varies considerably across existing studies. Many prior investigations have used reinsertion within 48–96 h as a primary endpoint to reflect procedural safety (4, 11), whereas others have focused on successful decannulation during hospitalization or at follow-up as a measure of overall clinical outcome (12, 13). Our study separates these dimensions by defining decannulation failure as a pathway-dependent process outcome and early reinsertion within 72 h as a safety outcome. This distinction may improve interpretability by aligning each outcome with its underlying clinical construct, particularly in studies comparing different decannulation pathways. Notably, in our study, all patients requiring reinsertion ultimately had a tracheostomy tube at hospital discharge, suggesting that early reinsertion may represent a marker of sustained decannulation unfitness rather than a transient event.

Importantly, the observed differences may be attributable not only to the decannulation pathway, but also to variations in clinical workflow and care processes, including monitoring intensity, rehabilitation priorities, and discharge practices. Although both pathways operated within a shared institutional framework, such pathway-level factors cannot be fully accounted for in a retrospective design and may contribute to the observed differences. Notably, the shorter observed time to decannulation is partly inherent to the pathway design, as the non-capping pathway intentionally bypasses the formal 24–48 h capping trial required in the conventional group. Accordingly, time to decannulation in this study should be interpreted as a process-related variation between the two clinical pathways rather than a direct measure of treatment effectiveness. Procedural variations, including tracheostomy tube type (metal versus plastic), were inherent to the pathways rather than determinants of treatment allocation and are unlikely to substantially influence overall decannulation success.

Our findings suggest that while conventional capping is traditionally regarded as a mandatory surrogate for airway patency and respiratory reserve (12), omission of a formal occlusion trial was not associated with higher rates of early reinsertion or decannulation failure, suggesting the feasibility of a direct-to-decannulation pathway. Notably, the rate of decannulation intolerance was comparable between groups (approximately 39%), suggesting that the non-capping pathway does not merely reflect a more permissive selection process but may indicate that bronchoscopic evaluation identifies “unready” candidates with the same sensitivity, while potentially reducing procedural delays.

From an analytical perspective, the interpretation of adjusted results also warrants careful consideration. Clinically, the non-capping group was inherently more complex (e.g., advanced age, higher frailty). Although IPTW-adjusted Firth regression was applied to account for baseline imbalances and showed a directionally consistent trend. Given the extremely low number of events, this weighted analysis should be considered exploratory, and emphasis should be placed on absolute event counts and the width of confidence intervals rather than statistical modeling alone.

Against this background, several potential mechanisms may explain the observed differences, although these remain speculative. The bronchoscopy-guided pathway enables direct visualization of airway anatomy, facilitating identification of structural abnormalities and more timely decision-making (8, 9, 14–16). In contrast, conventional capping may introduce additional resistive load and delay decannulation in patients with limited respiratory reserve (13, 17). Differences in airway management and exposure time may partly contribute to variation in infectious outcomes. While reduced artificial airway exposure may be one possible explanation (18, 19), this observation may also reflect potential biases, including unequal time at risk, the limited number of events and clinician-dependent infection ascertainment. Accordingly, these findings should be interpreted cautiously and do not establish a direct causal or mechanistic relationship.

Tracheostomy decannulation strategies exist along a spectrum, including stepwise capping protocols, direct decannulation, and hybrid approaches incorporating speaking valves or intermittent occlusion (20–22). The bronchoscopy-guided non-capping pathway represents one such approach emphasizing anatomical assessment over prolonged functional testing. However, this study was not designed to compare these strategies directly, and further studies are needed to define the optimal pathway for different patient populations.

## Limitations

Several limitations warrant consideration. First, its retrospective, non-randomized design introduces potential selection bias and residual confounding despite statistical adjustment. A significant limitation is the lack of objective metrics for cough strength, secretion volume beyond suction frequency, and formal swallow assessments, which are critical determinants of decannulation readiness. These unmeasured factors may lead to residual confounding. Therefore, residual confounding related to unmeasured or imperfectly measured readiness factors cannot be excluded. Second, the modest sample size and low number of events limit precision and increase susceptibility to model instability. Third, as a single-center study, the generalizability may be constrained by local institutional practices and resource availability, including potential bronchoscopy operator dependence. Finally, long-term follow-up and patient-centered functional measures were not assessed. Future prospective, multicenter studies incorporating standardized readiness assessments are needed to validate these findings and refine patient selection.

## Conclusion

In summary, a bronchoscopy-guided non-capping pathway was associated with a shorter time to decannulation and a lower number of infectious events, without an apparent increase in short-term safety events. However, these findings should be interpreted cautiously given differences in pathway structure, potential imprecision of statistical estimates, and the observational study design. This approach may represent a feasible alternative pathway in selected patients, warranting further validation in prospective multicenter studies.

## Data Availability

The datasets generated and analyzed in the current study are not publicly available due to institutional data protection policies but are available from the corresponding author on reasonable request. Requests to access the datasets should be directed to Huihua Hong, hhjoe@zcmu.edu.cn.
