# Supplementary material for: Bronchoscopy-guided non-capping decannulation pathway versus conventional capping trial in patients with prolonged tracheostomy: a retrospective comparative cohort study
Source: Front Med (Lausanne). 2026 May 15;13:1825058. doi: 10.3389/fmed.2026.1825058 (PMC13219283; doi:10.3389/fmed.2026.1825058)
Supplement: Supplementary file 1 [file Supplementary_file_1.docx]

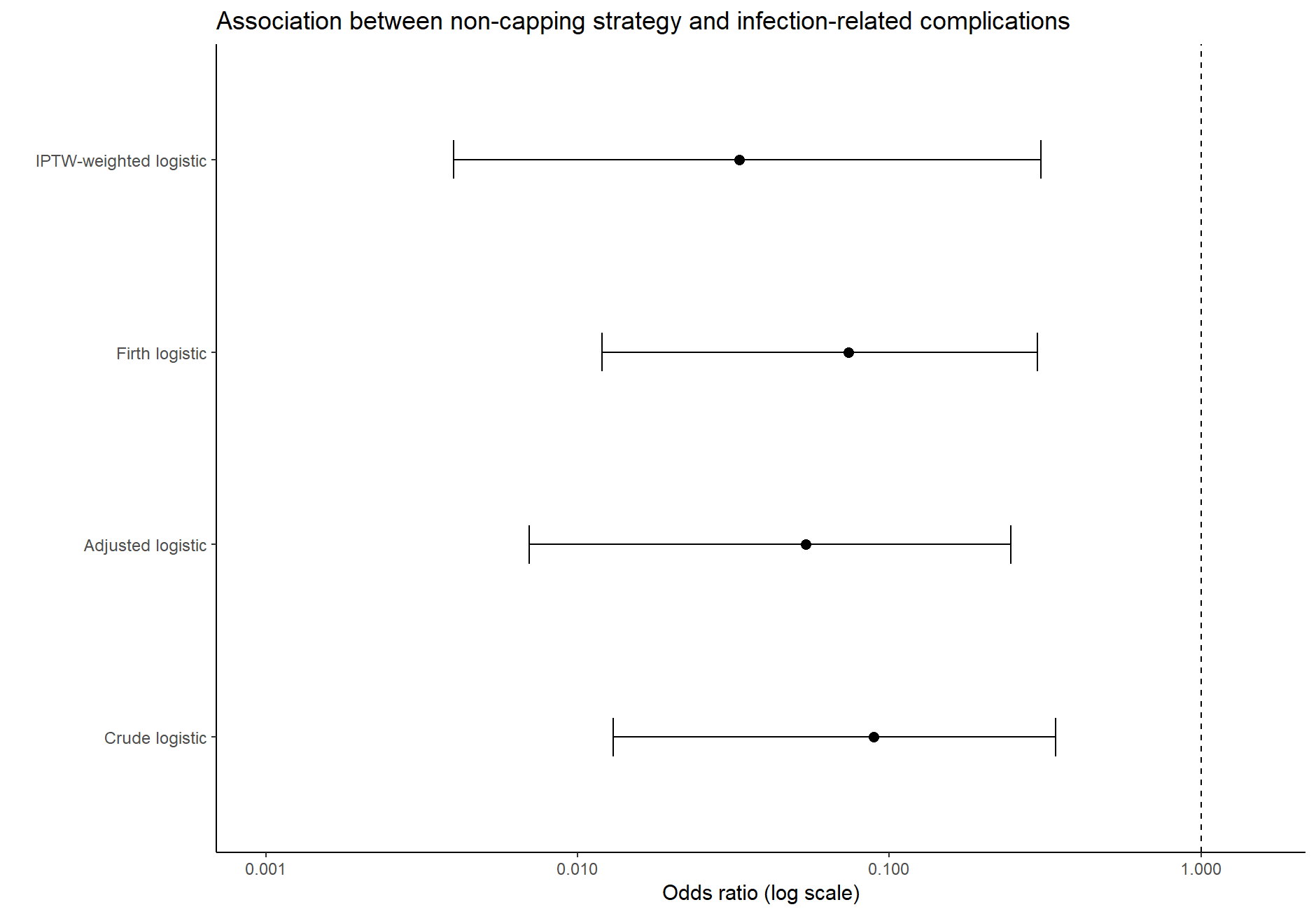


**Supplementary Figure 1. Forest plot summarizing the association between decannulation pathway and infectious complications across multiple analytic models.** The plot presents odds ratios (ORs) with 95% confidence intervals for the non-capping pathway compared with the conventional capping trial derived from crude logistic regression, multivariable-adjusted logistic regression, Firth’s penalised logistic regression, and **IPTW-weighted logistic regression** analyses. Across all models, the non-capping pathway showed lower observed infectious event rates.
