# Supplementary material for: Bronchoscopy-guided non-capping decannulation pathway versus conventional capping trial in patients with prolonged tracheostomy: a retrospective comparative cohort study
Source: Front Med (Lausanne). 2026 May 15;13:1825058. doi: 10.3389/fmed.2026.1825058 (PMC13219283; doi:10.3389/fmed.2026.1825058)
Supplement: Supplementary file 2 [file Supplementary_file_2.docx]

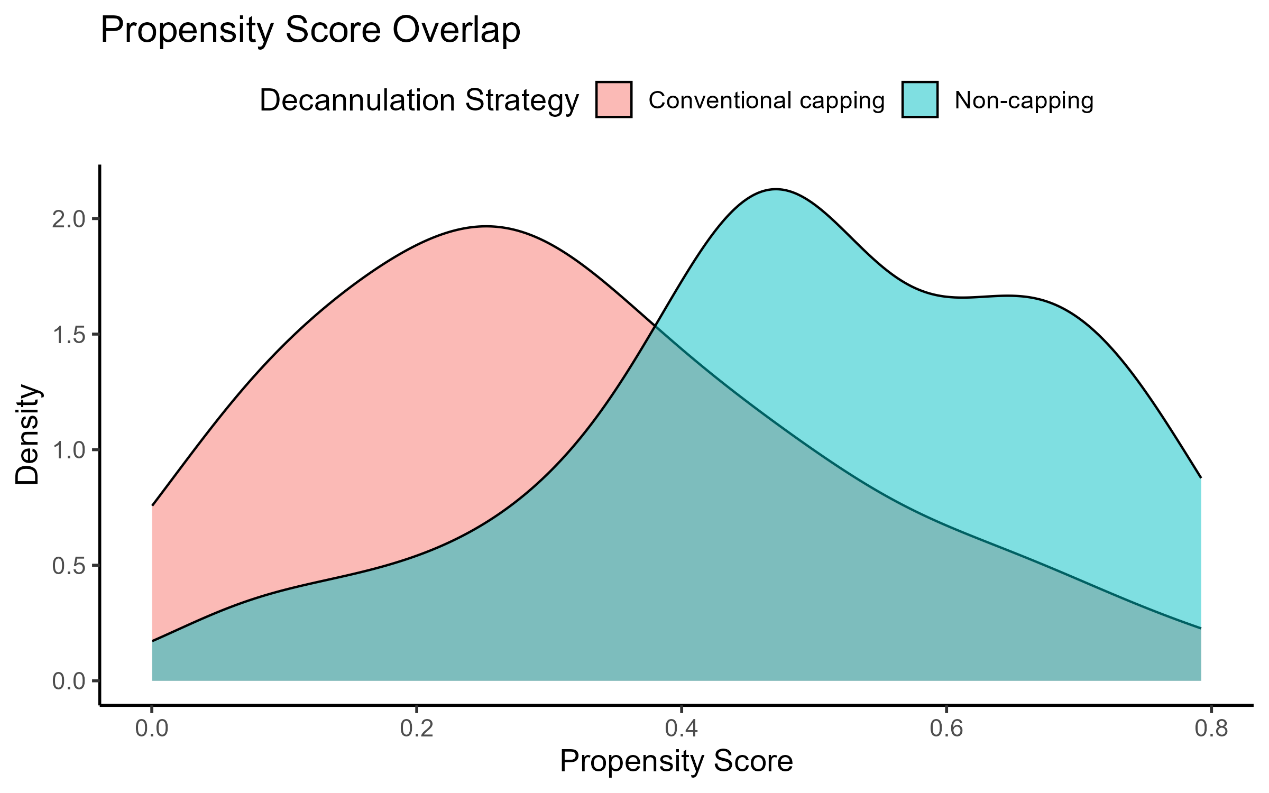


**Supplementary Figure 2. Propensity score overlap before and after weighting.** Density plot illustrating the distribution of estimated propensity scores for patients in the conventional capping (red) and non-capping (blue) groups. The substantial overlapping area confirms adequate common support between the two cohorts, methodologically justifying the use of inverse probability of treatment weighting (IPTW).
