# Supplementary material for: Bronchoscopy-guided non-capping decannulation pathway versus conventional capping trial in patients with prolonged tracheostomy: a retrospective comparative cohort study
Source: Front Med (Lausanne). 2026 May 15;13:1825058. doi: 10.3389/fmed.2026.1825058 (PMC13219283; doi:10.3389/fmed.2026.1825058)
Supplement: Supplementary file 3 [file Table_1.docx]

**Supplementary Table 1. Multivariable and sensitivity analyses evaluating the association between decannulation pathway and infectious complications.**

| Model | OR | CI | p_value |
| --- | --- | --- | --- |
| Crude logistic regression | 0.089 | 0.013–0.341 | 0.002 |
| Adjusted logistic regression | 0.054 | 0.007–0.245 | <0.001 |
| Firth penalized logistic regression | 0.074 | 0.012–0.299 | <0.001 |
| IPTW-weighted logistic regression | 0.033 | 0.004–0.307 | 0.003 |

**Note:** Results are presented as odds ratios (ORs) with 95% confidence intervals (CIs) for the non-capping pathway compared with the conventional capping trial. Four analytic approaches were applied: crude logistic regression, multivariable-adjusted logistic regression (adjusting for age, hemoglobin, and GCS-M score), Firth’s penalised logistic regression, and IPTW-weighted logistic regression. The IPTW analyses utilized truncated stabilized weights derived from a propensity score model incorporating age, hemoglobin, GCS-M, COPD, and pulmonary infection to further address confounding by indication. Across all analytic approaches, the non-capping pathway consistently showed lower observed infectious event rates across models.
