# Supplementary material for: Bronchoscopy-guided non-capping decannulation pathway versus conventional capping trial in patients with prolonged tracheostomy: a retrospective comparative cohort study
Source: Front Med (Lausanne). 2026 May 15;13:1825058. doi: 10.3389/fmed.2026.1825058 (PMC13219283; doi:10.3389/fmed.2026.1825058)
Supplement: Supplementary file 4 [file Table_2.docx]

**Supplementary Table 2. Complete covariate balance before and after inverse probability of treatment weighting (IPTW)**

| **Covariates** | **Unweighted Conventional Capping (Mean)** | **Unweighted Non-capping (Mean)** | **Unweighted SMD** | **Weighted Conventional Capping (Mean)** | **Weighted Non-capping (Mean)** | **Weighted SMD** |
| --- | --- | --- | --- | --- | --- | --- |
| **Age (years)** | 69.00 | 73.95 | 0.491 | 70.82 | 71.01 | 0.018 |
| **Hemoglobin (g/L)** | 100.50 | 91.95 | 0.592 | 96.98 | 98.27 | 0.089 |
| **Glasgow Coma Scale – Motor** | 5.53 | 5.90 | 0.527 | 5.67 | 5.75 | 0.111 |
| **COPD (%)** | 17.6 | 19.0 | 0.036 | 22.2 | 18.8 | 0.087 |
| **Pulmonary infection (%)** | 88.2 | 90.5 | 0.073 | 87.9 | 90.6 | 0.090 |

Notes: The table details the unweighted and weighted means, as well as the absolute standardised mean differences (SMDs), for all covariates included in the propensity score model before and after the application of truncated stabilised weights. (Notes: SMD, standardized mean difference. For categorical variables such as COPD and pulmonary infection, the values represent the percentage of patients with the condition.)
